# Underfilling decreases left ventricular function in pulmonary arterial hypertension

**DOI:** 10.1007/s10554-020-02143-6

**Published:** 2021-01-27

**Authors:** Hannah Sjögren, Barbro Kjellström, Anna Bredfelt, Katarina Steding-Ehrenborg, Göran Rådegran, Roger Hesselstrand, Håkan Arheden, Ellen Ostenfeld

**Affiliations:** 1 Department of Clinical Sciences Lund, Clinical Physiology and Skåne University Hospital, Lund University, Lund, Sweden; 2 Cardiology Unit, Department of Medicine, Karolinska Institutet, Stockholm, Sweden; 3 Department of Health Sciences, Physiotherapy, Lund University, Lund, Sweden; 4 Department of Clinical Sciences Lund, Cardiology, and the Section for Heart Failure and Valvular Disease, Skåne University Hospital, Lund University, Lund, Sweden; 5 Department of Clinical Sciences Lund, Rheumatology, The Clinic for Rheumatology, Skåne University Hospital, Lund University, Lund, Sweden; 6 Department of Clinical Physiology, Skåne University Hospital, Lund, Sweden

**Keywords:** Pulmonary hypertension, Left ventricular dysfunction, Left atrial volume, Feature tracking strain, Cardiac magnetic resonance imaging

## Abstract

To evaluate the association between impaired left ventricular (LV) longitudinal function and LV underfilling in patients with pulmonary arterial hypertension (PAH). Thirty-nine patients with PAH and 18 age and sex-matched healthy controls were included. LV volume and left atrial volume (LAV) were delineated in short-axis cardiac magnetic resonance (CMR) cine images. LV longitudinal function was assessed from atrio-ventricular plane displacement (AVPD) and global longitudinal strain (GLS) was assessed using feature tracking in three long-axis views. LV filling was assessed by LAV and by pulmonary artery wedge pressure (PAWP) using right heart catheterisation. Patients had a smaller LAV, LV volume and stroke volume as well as a lower LV-AVPD and LV-GLS than controls. PAWP was 6 [IQR 5––9] mmHg in patients. LV ejection fraction did not differ between groups. LV stroke volume correlated with LV-AVPD (r = 0.445, p = .001), LV-GLS (r = − 0.549, p < 0.0001) and LAVmax (r = .585, p < 0.0001). Furthermore, LV-AVPD (r = .598) and LV-GLS (r = − 0.675) correlated with LAVmax (p < 0.0001 for both). Neither LV-AVPD, LV-GLS, LAVmax nor stroke volume correlated with PAWP. Impaired LV longitudinal function was associated with low stroke volume, low PAWP and a small LAV in PAH. Small stroke volumes and LAV, together with normal LA pressure, implies that the mechanism causing reduced LV longitudinal function is underfilling rather than an intrinsic LV dysfunction in PAH.

## Introduction

Pulmonary arterial hypertension (PAH) is a progressive disease with high mortality [1–3] and patients with PAH associated to systemic sclerosis (APAH-SSc) have an even more ominous prognosis than patients with the idiopathic or familiar form of PAH (IPAH) [1]. The pathophysiological and prognostic focus has mainly been on the right ventricle (RV), since the increased right-sided afterload in PAH leads to RV failure [4, 5]. Less focus has been on the left ventricle (LV), although LV function is reduced despite preserved LV ejection fraction (LVEF) [6–10].

LV global longitudinal strain (GLS) has been shown to be of prognostic value in SSc, APAH-SSc and PAH [8, 9, 11, 12]. Furthermore, in patients with pulmonary hypertension, left atrioventricular plane displacement (AVPD) and longitudinal contribution to stroke volume are reduced, despite preserved LVEF [6]. While longitudinal contribution to stroke volume measures the volumetric effects of longitudinal pumping, the measure carries no information on myocardial contractile function. Strain, on the other hand, can be used as a surrogate measure of the myocardial contraction [13].

The mechanisms of the altered left sided longitudinal functional measures in PAH are, however, not fully understood. One plausible hypothesis is that the LV is actually healthy, even if ‘understimulated’ owing to the limited flow through the pulmonary circulation [14]. If so, the altered LV longitudinal function is not mechanistically a pathological reduction due to a true myocardial dysfunction, but a natural consequence of underfilling [15, 16]. In left-sided heart failure, enlarged left atrial (LA) volumes are indicative of impaired LV filling due to increased LV pressure [17], and, oppositely, decreased LA volumes might hence be a measure of underfilling of the LV [18].

Therefore, the aim of the present study was to evaluate if there is an association between LV longitudinal function and LV underfilling in patients with PAH, and if this could provide a mechanistic explanation for the reduced LV longitudinal function. To this end, we assessed LV function by LV-AVPD and LV-GLS measurements as well as left-sided filling by LA volumes and stroke volume, and we related these measures to LA pressure estimated invasively by pulmonary artery wedge pressure (PAWP).

## Materials and methods

### Study population

Adult patients with PAH (IPAH or APAH-SSc) who underwent a clinically motivated cardiac magnetic resonance (CMR) examination and right heart catheterisation in 2003–2015 were retrospectively included (Table 1). Between 2003 and 2015, 182 patients were diagnosed with PAH at our centre. Of these, all but three underwent a right heart catheterisation and 117 were examined with CMR. Of those, 44 patients with IPAH and 29 patients with APAH-SSc were relevant for inclusion in this study. Patients were excluded from analysis (n = 34) if presence of atrial fibrillation or inadequate CMR image quality. Hence, images from 39 patients were used in the present study. Healthy controls matched for sex and age and included in previous publications from our group were used for comparison [19, 20].

**Table 1:** Study participants’ baseline characteristics

	PAH n = 39		Controls n = 18		*p* value
Sex, women/men (%)	26/13	(67/33)	11/7	(61/39)	0.795
Age (years)	61	[37–72]	51	[43–55]	0.067
BSA (m^2^)	1.8	[1.7–2.1]	1.9	[1.7–2.0]	0.655
NT-proBNP (ng/l)	1689	[262–4277]			
De novo	19	(49)			
Time diagnosis to MR (days)	2	[0–289]			
Time RHC to MR (days)	1	[1–2]			
PAH-dedicated medication at CMR	26	(67)			
Right heart catheterisation					
RAP (mmHg)	5	[3–9]			
mPAP (mmHg)	43	[39–55]			
PAWP (mmHg)	6	[5–9]			
PVR (WU)	7.9	[5.7–11.5]			
Cardiac magnetic resonance					
Heart rate (bpm)	77	[70–92]	59	[54–68]	*< 0.0001*
CO (l/min)	4.9	[3.9–5.6]	6.3	[4.8–6.5]	*0.022*
CI (l/min/m^2^)	2.6	[2.2–3.3]	3.1	[2.9–3.5]	*0.039*
LVEDV (ml/m^2^)	61	[51–74]	89	[77–98]	* < 0.0001*
LVESV (ml/m^2^)	29	[21––34]	38	[30–41]	*0.001*
LVSV (ml/m^2^)	36	[28–43]	51	[47–55]	* < 0.0001*
LVEF (%)	57	[48–62]	58	[55–64]	0.250
RVEDV (ml/m^2^)	113	[98–152]	92	[77–105]	*0.0005*
RVESV (ml/m^2^)	79	[54–107]	38	[29–49]	*< 0.0001*
RVSV (ml/m^2^)	40	[37–48]	52	[47–56]	*< 0.0001*
RVEF (%)	33	[26–46]	56	[53–63]	* < 0.0001*
LAVmin (ml/m^2^)	17	[14–20]	21	[19–23]	*0.022*
LAVmax (ml/m^2^)	32	[26–42]	50	[40–53]	*< 0.0001*
LAEV (ml/m^2^)	15	[11–20]	28	[21–33]	*< 0.0001*
LAEF (%)	43	[36–51]	56	[47–60]	*0.006*
LV-AVPD (mm)	11.6	[8.7–13.7]	16.1	[15.1–17.2]	*< 0.0001*
LV-GLS (%)	− 14.9	[− 16.7 to − 12.9]	− 18.4	[− 19.3 to − 17.1]	* < 0.0001*

Data expressed as median and interquartile range [IQR] or in absolute number and proportion in parenthesis. *BSA* body surface area; *CI* cardiac index; *CMR* cardiac magnetic resonance; *CO* cardiac output; *De novo* new diagnosis, untreated at time of investigation; *LAEF* left atrial emptying fraction; *LAEV* left atrial emptying volume indexed to body surface area; *LAVmax* left atrial maximum volume indexed to body surface area; *LAVmin* left atrial minimum volume indexed to body surface area; *LV-AVPD* left ventricular atrioventricular plane displacement; *LVEDV* left ventricular end-diastolic volume indexed to body surface area; *LVEF* left ventricular ejection fraction; *LVESV* left ventricular end-systolic volume indexed to body surface area; *LV-GLS* left ventricular global longitudinal strain; *LVSV* left ventricular stroke volume indexed to body surface area; *mRAP* mean right atrial pressure; *mPAP* mean pulmonary arterial pressure; *PAH* pulmonary arterial hypertension; *PAWP* pulmonary artery wedge pressure; *PVR* pulmonary vascular resistance; *RHC* right heart catheterisation; *RVEDV* right ventricular end-diastolic volume indexed to body surface area; *RVEF* right ventricular ejection fraction; *RVESV* right ventricular end-systolic volume indexed to body surface area; *RVSV* right ventricular stroke volume indexed to body surface area. Significant p-values marked with italics

### Right heart catheterisation

Right heart catheterisation in conjunction with CMR (median time between right heart catheterisation and CMR 1 [IQR 1–2] day) was performed under local anaesthesia on all patients, in a supine position using a triple-lumen 7-F Swan Ganz catheter. Measurements included mean right atrial pressure (mRAP), mean pulmonary artery pressure (mPAP), PAWP, cardiac output (CO) measured by thermodilution, and pulmonary vascular resistance (PVR) (calculated as (mPAP-PAWP)/CO). N-terminal pro-brain natriuretic peptide (NT-proBNP) was acquired from venous blood samples in conjunction with the examinations.

### Image acquisition

CMR was performed using 1.5T Philips Achieva (Philips Medival System, Best, The Netherlands) with a cardiac synergy coil and with participants in a supine position. Balanced steady-state free precession cine images were acquired with ECG-triggered imaging during end-expiratory apnoea (typically for 15 s at each acquisition). Short-axis images covering the entire heart were acquired. Long axis images were acquired in the LV two-chamber view, three-chamber view and four-chamber view. Typical imaging parameters were: slice thickness of 8 mm with no slice gap, temporal resolution of 47 ms reconstructed in 30 times phases per cardiac cycle, repetition time 3 ms, echo time 1.4 ms and flip angle 60°.

### Image analysis

**Fig. 1:**
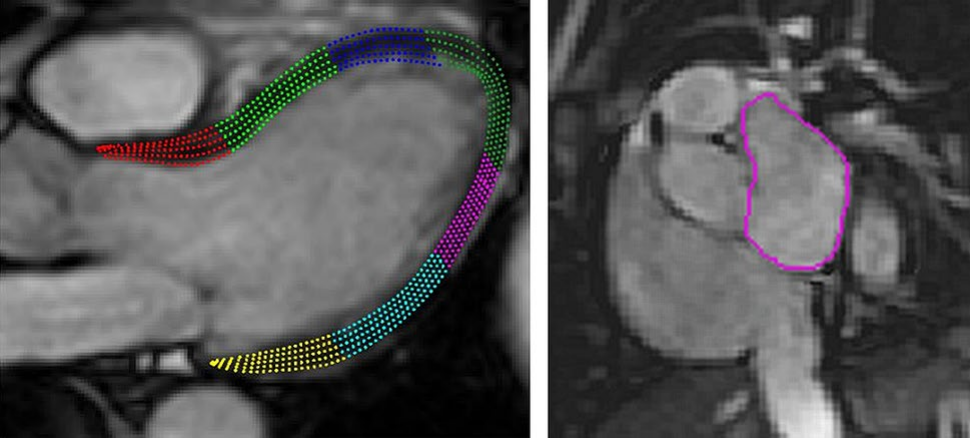
Example of myocardial tracing in 3-chamber view (left) and delineation of left atrium (right) in a patient with associated pulmonary arterial hypertension owing to systemic sclerosis

All image analysis of baseline data, including LA volume measurements, LV-AVPD and LV-GLS (Fig. 1), was performed in the freely available software Segment 2.2 R6887 (Medviso, Lund, Sweden; [21]). LV end-diastolic volume, end-systolic volumes and stroke volumes were obtained from short-axis images by delineations of the ventricular epicardial and endocardial borders. Stroke volume was calculated as endocardial end-systolic volume subtracted from end-diastolic volume. LVEF was calculated as stroke volume divided by end-diastolic volume.

The endocardium of the LA was manually delineated in a stack of short-axis views with the maximum volume (LAVmax) at ventricular end systole, and minimum volume (LAVmin) at ventricular end diastole (Fig. 1). Pulmonary veins were excluded, and LA appendage included in the volumes. LA emptying volume (LAEV) was computed as LAVmax – LAVmin.

LV-AVPD was measured at eight points in three long-axis views by subtracting the AV plane position in end-systole from that in end diastole, as previously described [6, 22, 23].

All cardiac volumes were indexed to body surface area.

### Strain analysis

Global longitudinal strain was analysed in three long-axis cine images (2-, 3-, and 4-chamber view) using feature tracking. Delineations along the epi- and endocardial borders in the myocardium were drawn manually in a single frame in end diastole, after which the software calculated the myocardial movement throughout the cardiac cycle using a vector-based algorithm. Manual adjustments of the delineations were applied in end diastole with new propagation if contours did not comply properly through systole (Fig. 1).

Peak GLS was defined as *GLS* (%) = (*ML*_*s*_*− ML*_*d*_)/*ML*_*d*_ × 100; with ML_s_ representing the myocardial length at end systole and ML_d_ the myocardial length at end diastole [24]. LV was assessed in the 17-segment model according to guidelines [24].

### Statistical methods

Statistical analysis was performed using SPSS (IBM Corp. Released 2017. IBM SPSS Statistics for Macintosh, Version 25.0. Armonk, NY: IBM Corp.). Continuous data is presented as median with interquartile range [IQR] according to normal distribution tested visually by histograms. Categorical data is presented in absolute numbers and proportion in percentage. Comparisons between patients with PAH and controls as well as between patients with IPAH and APAH-SSc were performed using independent samples Mann–Whitney U tests or Fisher’s exact test. A multivariate post-hoc test including Box’s test of equality of covariance matrices and Levene’s test of equality of error variance was performed. Pearson correlation (r) was used for correlations. A two-sided p value < 0.05 was considered statistically significant.

## Results

Thirty-nine patients with PAH (26 women; median age 61 [IQR 37–72] years; IPAH = 20 and APAH-SSc = 19) and 18 controls (11 women; age 51 [43–55] years) matched for age and sex were included (Tables 1 and 2). Nineteen patients (49%) were de novo investigations.

**Table 2:** Baseline characteristics for the PAH subgroups idiopathic (IPAH) and associated to systemic sclerosis (APAH-SSc)

	IPAH n = 20	APAH-SSc n = 19	*p* value
Sex, women/men (%)	12/8 (60/40)	14/5 (74/26)	0.365
Age (years)	45 [29–73]	65 [58–72]	0.061
BSA (m^2^)	1.8 [1.7–2.1]	1.7 [1.6–1.9]	0.224
NT-proBNP (ng/l)	1689 [693–4277]	1866 [225–4567]	0.612
De novo	10 (50)	9 (47)	0.870
Time diagnosis to CMR (days)	1 [0–8]	190 [1–863]	0.104
Time RHC to CMR (days)	1 [0–5]	1 [1]	0.090
PAH-dedicated medication	11 (55)	15 (79)	0.176
*Right heart catheterization*			
RAP (mmHg)	7 [4–11]	4 [2–8]	0.123
mPAP (mmHg)	50 [40–59]	40 [29–44]	*0.005*
PAWP (mmHg)	8 [5–11]	6 [3–8]	0.051
PVR (WU)	10.1 [7.9–14.8]	6.0 [4.9–7.7]	*0.0007*
Cardiac magnetic resonance			
Heart rate (bpm)	75 [70–88]	82 [73–94]	0.322
CO (l/min)	4.7 [3.4–5.2]	5.0 [4.1–6.4]	0.283
CI (l/min/m^2^)	2.5 [2.0–3.1]	2.8 [2.2–3.7]	0.141
LVEDV (ml/m^2^)	59 [49–75]	65 [52–74]	0.627
LVESV (ml/m^2^)	30 [21–34]	29 [21–35]	0.749
LVSV (ml/m^2^)	31 [27–37]	36 [28–45]	0.247
LVEF (%)	56 [47–61]	58 [51–62]	0.411
RVEDV (ml/m^2^)	113 [99–151]	113 [86–159]	0.632
RVESV (ml/m^2^)	83 [57–100]	74 [50–109]	0.627
RVSV (ml/m^2^)	42 [36–53]	39 [37–46]	0.457
RVEF (%)	32 [25–46]	34 [28–46]	0.780
LAVmin (ml/m^2^)	17 [12–23]	17 [14–19]	0.945
LAVmax (ml/m^2^)	32 [27–42]	32 [25–42]	0.923
LAEV (ml/m^2^)	14 [10–18]	18 [12–21]	0.380
LAEF (%)	41 [36–49]	44 [36–0.52]	0.531
LV-AVPD (mm)	11.1 [8.2–14.3]	12.0 [8.7–13.4]	0.681
LV-GLS (%)	− 14.6 [− 16.7 to − 13.0]	− 15.7 [− 16.9 to − 12.2]	0.588

Data expressed as median and interquartile range [IQR] or in absolute number and proportion in parenthesis. *BSA* body surface area, *CI* cardiac index, *CMR* cardiac magnetic resonance, *CO* cardiac output, *De novo* new diagnosis, untreated at time of investigation, *LAEF* left atrial emptying fraction, *LAEV* left atrial emptying volume indexed to body surface area, *LAVmax* left atrial maximum volume indexed to body surface area, *LAVmin* left atrial minimum volume indexed to body surface area, *LV-AVPD* left ventricular atrioventricular plane displacement, *LVEDV* left ventricular end-diastolic volume indexed to body surface area, *LVEF* left ventricular ejection fraction, *LVESV* left ventricular end-systolic volume indexed to body surface area, LV-GLS, left ventricular global longitudinal strain. LVSV, left ventricular stroke volume indexed to body surface area; mRAP, mean right atrial pressure, *mPAP* mean pulmonary arterial pressure, *PAH* pulmonary arterial hypertension, *PAWP* pulmonary artery wedge pressure, *PVR* pulmonary vascular resistance, *RHC* right heart catheterisation, *RVEDV* right ventricular end-diastolic volume indexed to body surface area, *RVEF* right ventricular ejection fraction, *RVESV* right ventricular end-systolic volume indexed to body surface area, *RVSV* right ventricular stroke volume indexed to body surface area. Significant p-values marked with italics

**Table 3:** Correlations between left ventricular and left atrial measures in the whole population (n = 57)

	LAVmin	LAVmax	LAEV	LV-AVPD	LV-GLS
LAVmax	0.662	–	–	–	
	< 0.0001				
LAEV	0.040	0.775	–	–	
	0.770	*< *0.0001			
LV-AVPD	0.112	0.589	0.691	–	
	0.405	*< *0.0001	*< *0.0001		
LV-GLS	– 0.364	– 0.675	– 0.593	– 0.527	
	0.005	*< *0.0001	*< *0.0001	*< *0.0001	
SV	0.258	0.688	0.699	0.683	– 0.667
	0.052	*< *0.0001	*< *0.0001	*< *0.0001	< 0.0001

R-values with p-values below. *LAEV* left atrial emptying volume indexed to body surface area, *LAVmax* left atrial maximum volume indexed to body surface area, *LAVmin* left atrial minimum volume indexed to body surface area, *LV-AVPD* left ventricular atrioventricular plane displacement, *LV-GLS* left ventricular global longitudinal strain, *SV* stroke volume indexed to body surface area. Significant p-values marked with italics

**Table 4:** Correlations between left ventricular and left atrial measures in the PAH-group (n = 39)

	LAVmin	LAVmax	LAEV	LV-AVPD	LV-GLS
LAVmax	0.726	–	–	–	
	*< *0.001				
LAEV	– 0.175	0.550	–	–	
	0.287	*< *0.001			
LV-AVPD	– 0.008	0.364	0.531	–	
	0.960	0.023	0.001		
LV-GLS	– 0.170	– 0.422	– 0.397	– 0.320	
	0.300	0.008	0.012	0.047	
SV	0.182	0.465	0.445	0.538	– 0.554
	0.268	0.003	0.005	*<*0.0001	< 0.0001

R-values with p-values below. *LAEV* left atrial emptying volume indexed to body surface area, *LAVmax* left atrial maximum volume indexed to body surface area, *LAVmin* left atrial minimum volume indexed to body surface area, *LV-AVPD* left ventricular atrioventricular plane displacement, *LV-GLS* left ventricular global longitudinal strain, *SV* stroke volume indexed to body surface area. Significant p-values marked with italics

Patients with PAH had a smaller LA volume, LV volume, stroke volume and CO than controls (Table 1). Patients with PAH also had a lower LV-AVPD and LV-GLS compared with controls. LVEF did not differ between groups. These results were consistent in the multivariate post-hoc test. PAWP was normal with a median of 6 [IQR 5–9] mmHg in patients.

In the whole study population, stroke volume correlated with LV-AVPD, LV-GLS, LAVmax and LAEV (Table 3, p < 0.0001 for all). LV-GLS and LV-AVPD correlated with LAVmax, LAVmin and LAEV (Table 3).

**Fig. 2:**
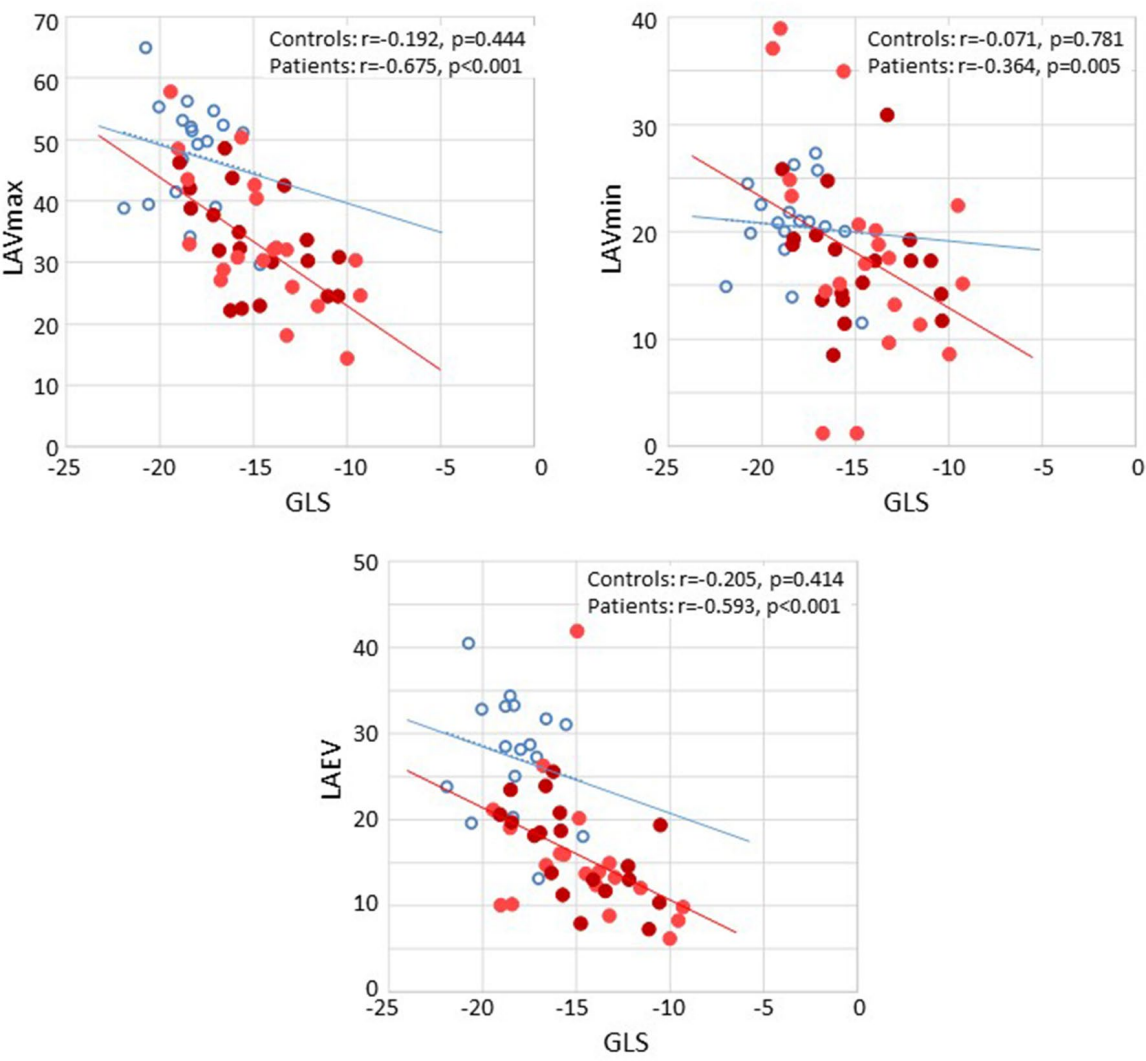
Correlation between peak systolic left ventricular global longitudinal strain (GLS) and left atrial (LA) volumes in healthy controls (blue circles) and patients with pulmonary arterial hypertension (red filled circles; light red are idiopathic and dark red are associated to systemic sclerosis). *LAVmax* LA maximum volume indexed to body surface area, *LAVmin* LA minimum volume indexed to body surface area, *LAEV* LA emptying volume indexed to body surface area

**Fig. 3:**
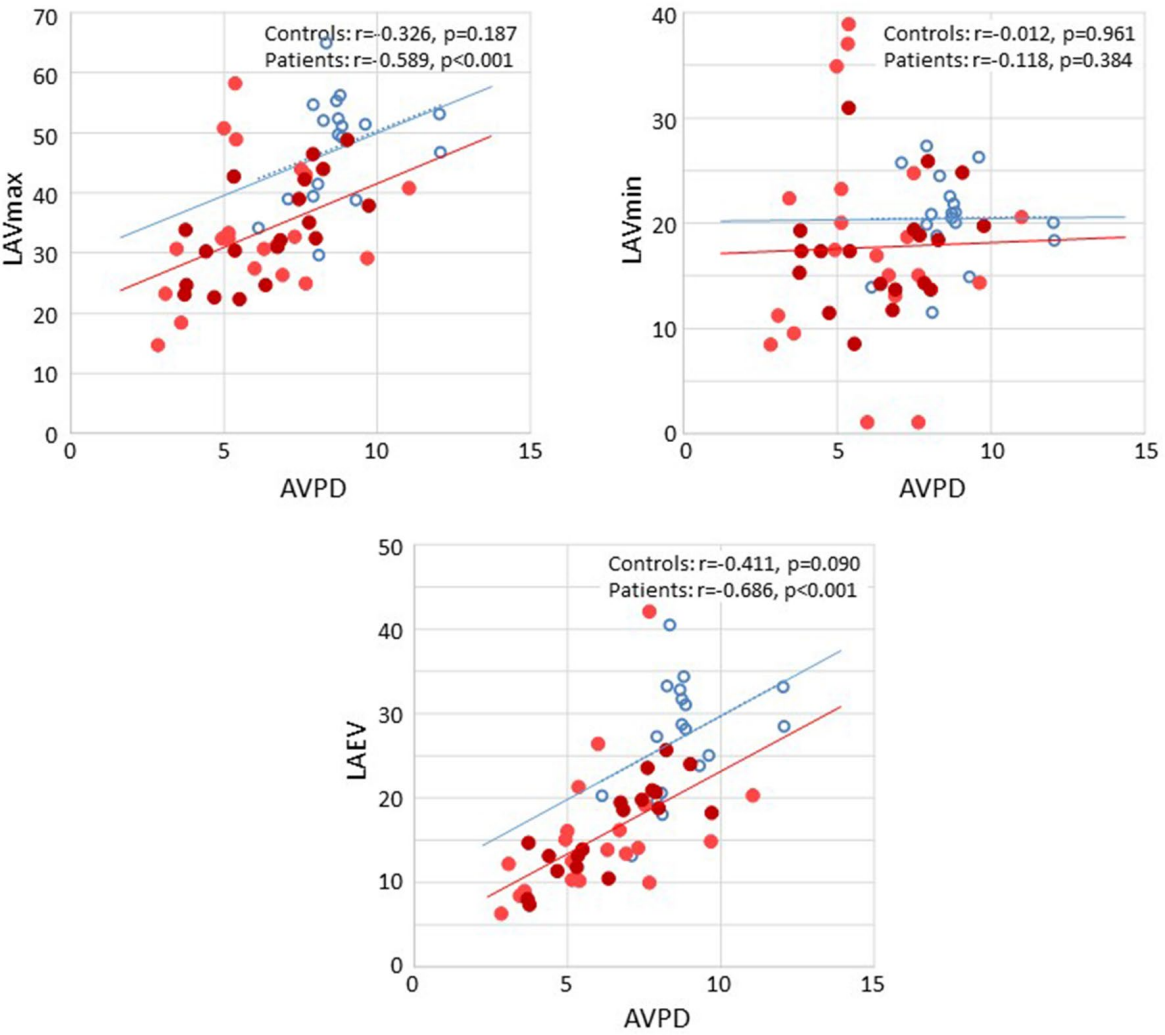
Correlation between peak systolic left ventricular atrioventricular plane displacement (AVPD) and left atrial (LA) volumes in healthy controls (blue circles) and patients with pulmonary arterial hypertension (red filled circles; light red are idiopathic and dark red are associated to systemic sclerosis). *LAVmax* LA maximum volume indexed to body surface area, *LAVmin* LA minimum volume indexed to body surface area, *LAEV* LA emptying volume indexed to body surface area

**Fig. 4:**
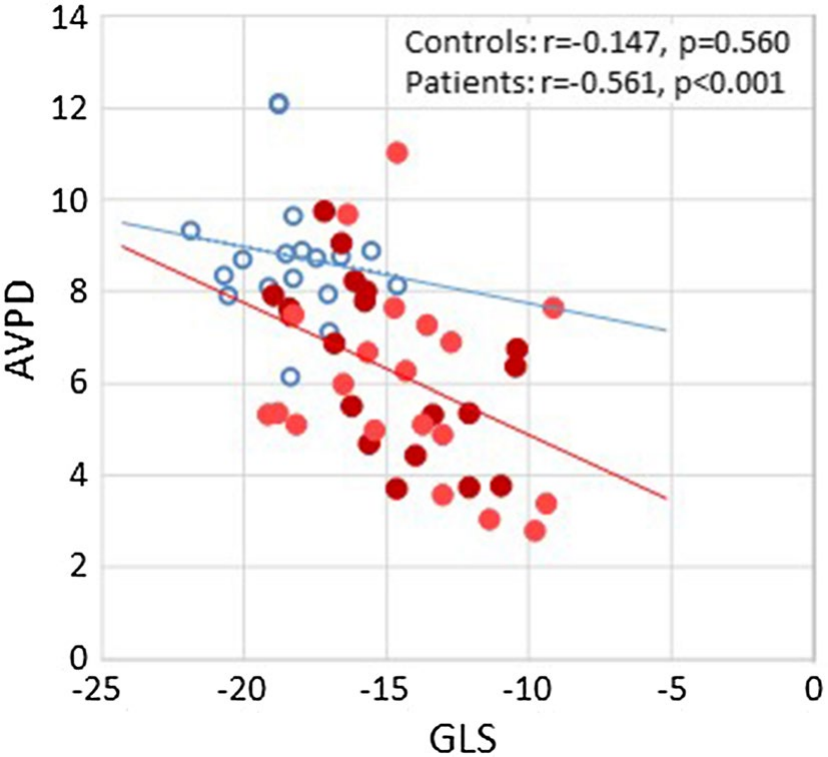
Correlation between peak systolic left ventricular global longitudinal strain (GLS), and atrioventricular plane displacement (AVPD) in healthy controls (blue circles) and patients with pulmonary arterial hypertension (red filled circles; light red are idiopathic and dark red are associated to systemic sclerosis). R^2^ = 0.02 in healthy controls and R^2^ = 0.278 in patients

In the PAH group, stroke volume correlated with LV-AVPD, LV-GLS, LAVmax and LAEV (Table 4). LV-GLS (Fig. 2) and LV-AVPD (Fig. 3) correlated with LAVmax, LAVmin and LAEV (Table 4) and LV-GLS correlated with LV-AVDP (Fig. 4).

PAWP and NT-proBNP did not correlate with stroke volume, LV-AVPD, LV-GLS, LAVmax or LAEV in patients (Table 5). Stroke volume, LV-GLS and LAVmax were associated with mPAP and PVR, while LV-AVPD was not (Table 5).

**Table 5:** Correlations between pulmonary artery wedge pressure and NT-proBNP to left ventricular and left atrial measures

	SV	LV-AVPD	LV-GLS	LAVmax	LAEV
PAWP	0.010	– 0.011	– 0.146	0.240	0.259
	0.955	0.948	0.390	0.153	0.122
NT-proBNP	0.006	– 0.191	– 0.010	– 0.137	0.079
	0.974	0.272	0.953	0.433	0.650
PVR	– 0.698	– 0.313	0.375	– 0.414	– 0.441
	*< *0.0001	0.059	0.022	0.011	0.006
mPAP	– 0.556	– 0.235	0.458	– 0.485	– 0.288
	0.0003	0.162	0.004	0.002	0.084

R-values with p-values below. *LAEV* left atrial emptying volume indexed to body surface area, *LAVmax* left atrial maximum volume indexed to body surface area, *LV-AVPD* left ventricular atrioventricular plane displacement, *LV-GLS* left ventricular global longitudinal strain, *mPAP* mean pulmonary arterial pressure, *NT-proBNP* N-terminal pro brain natriuretic peptide, *PAWP* pulmonary artery wedge pressure, *PVR* pulmonary vascular resistance, *SV* stroke volume indexed to body surface area. Significant p-values marked with italics

In the controls, there was no correlation between stroke volume and LV-AVPD, LV-GLS, LAVmax, LAVmin or LAEV (p = 0.097, p = 0.942, p = 0.111, p = 0.365 and p = 0.137; respectively). Further, LV-GLS (Fig. 2) and LV-AVPD (Fig. 3) did not correlate with LAVmax (p = 0.187 and p = 0.444) and neither did LV-GLS correlate with LV-AVDP (Fig. 4) in healthy participants.

For the subgroups IPAH and APAH-SSc there were no differences in stroke volume, LA or LV measures (Table 2), even if patients with IPAH had higher mPAP and higher PVR than patients with APAH-SSc. CO, PAWP and NT-proBNP and PAH-dedicated medication at CMR examination did not differ between subgroups (Table 2). These results were consistent in the multivariate post-hoc test.

## Discussion

Reduced longitudinal function was associated with smaller LV stroke volumes and LA volumes at normal LA pressures in patients with PAH. This implies that the mechanism behind the reduced longitudinal function is an underfilling of the left heart rather than an intrinsic LV dysfunction. This leads to reduced stroke volumes which are likely not affected by the pressures themselves since stroke volume, right atrial pressure, LA measures and LV measures did not differ between patients with IPAH and APAH-SSc, despite higher pulmonary artery pressure and PVR in IPAH.

The smaller LV volumes among patients with PAH compared with controls are in concordance with previous studies [4, 25]. There are several suggested explanations to the smaller LV volumes. For one, smaller LV volumes have been described as being a consequence of septal bowing in the RV pressure overloaded situation [26]. However, it has also been suggested that the low LV preload and relative underfilling in itself can result in an impaired LV relaxation pattern without abnormal septum or LV appearance [9, 14, 16]. The decreased LV volumes have also been proposed to be caused by increased PVR as this will reduce the blood flow to the left heart [25]. In our study, patients with IPAH had higher pulmonary artery pressure and PVR than those with APAH-SSc, yet there were no differences in stroke volume, right atrial pressure, LA measures or LV measures between these subgroups. This infers that an increased afterload in the RV is not in itself the sole mechanism causing the small LV.

The decreased flow through the pulmonary circulation, indicated by the lower stroke volume and CO, in PAH compared with controls, is in agreement with previous studies [4, 15, 16, 25, 26]. In the situation of normal LV contractility and low supply of blood to the left side, the LV would suck in blood assiduously and the LA would reduce in size. When the LA cannot ‘feed’ the LV with volume, a reduction in LV volume is the result. As such, the reduced forward volumes through the high PVR decreases LA and LV volumes in patients with PAH [4, 25, 27]. Hence, this deconditioning of the myocardium might affect the measures of pump function [28–30]. The combination of low flow and small left-sided chambers will reduce the need for high contractile force on the left side, as an effect of the decreased myocardial stretch [10, 31]. Moreover, a reduced LV filling and concomitant reduced LV volume-loading have been shown to change the structure of the LV cardiomyocytes leading to an atrophied myocardium and decreased contractility [10, 29, 30, 32]. In the present study, the lower LV-GLS in patients with PAH compared with controls indicates, that the LV contractility was affected. The lower LV-GLS at preserved ejection fraction is in concordance with previous studies and has been associated with early mortality [9]. The LV-AVPD and LV-GLS values are even in parity with those in substantial LV heart failure. Should the decreased longitudinal measures in our study have been caused by an intrinsic LV dysfunction, one would expect to find increased PAWP and LA volumes. However, neither PAWP nor LA volumes were increased. On the contrary, both were within normal ranges, which would be expected in patients with PAH. This implies that the decreased longitudinal LV-AVPD and LV-GLS are not a ‘true’ dysfunction. Of note, structural changes related to decreased load of the LV have been described, such as in patients undergoing corrective surgery for transposition of the greater arteries [30].

The LV-AVPD was lower in patients with PAH compared with controls, despite normal LVEF. This can be explained by different mechanisms. First, the LV-AVPD is generated from the ventricular contraction. A reduced myocardial contractility and hence a lower GLS would intrinsically generate a lower AVPD. However, the r^2^ coefficient between LV-AVPD and LV-GLS was 0.278, which indicates that causes other than decreased GLS are more determinant for the reduction in LV-AVPD (Fig. 4). Secondly, the AV plane is a fibrous plate and as AVPD is decreased on the right side [6], this could cause the AVPD to be less agile on the left side. Thirdly, the AV plane works like a piston [33], being the major contributing factor in generating ventricular stroke volume [23]. The AV plane is also the major cause of atrial aspiration of blood from the pulmonary and caval veins [34]. When the supply of blood to the left side is restricted by obstruction in the pulmonary circulation, the AV plane cannot increase the filling by suction. Hence, the AVPD would be expected to be lower in the underfilling situation.

In systemic sclerosis (SSc), the autoimmune connective tissue disorder may involve the heart with cardiac fibrosis and PAH [35]. Patients with SSc have been shown to be prone to having myocardial fibrosis and could therefore be expected to have impaired myocardial stiffness, increased LV filling pressures and hence increased LA volumes. However, in a previous CMR study, patients with SSc did not have lower LV-GLS unless infarction or localised fibrosis was present [36]. Furthermore, low LV-GLS was shown to be due to PAH rather than SSc in itself [12]. In the present study, we did not find differences in LA volumes or LV measures between patients with IPAH and APAH-SSc. PAH-dedicated medication could influence PVR and mPAP values and patients with SSc might be prescribed PAH-dedicated medication owing to their cutaneous and vascular manifestations of SSc before PAH has developed. However, we did not find a significant difference in PAH-dedicated medication between the subgroups. The reduced stroke volume, LV-GLS and LAVmax were indeed associated with increased PVR and mPAP. Of note, despite higher PVR and mPAP in patients with IPAH, stroke volume and CO did not differ between the groups, which further supports the hypothesis, that the hypokinetic circulation and hence underfilling of the LV is similar in both groups. All these findings suggest that it is not likely that LV fibrosis is the mechanism behind the lower LV-GLS.

NT-proBNP is an established marker in risk assessment of patients with PAH [1] as well as in patients with congested left-sided heart failure [37]. We did not find any association between NT-proBNP and the left-sided measures of LV-AVPD, LV-GLS and LA volume, despite NT-proBNP being increased in both IPAH and APAH-SSc. This supports the notion that the elevated NT-proBNP is related to the right-sided stretch rather than it being an intrinsic LV dysfunction.

One might speculate that if the decreased LV longitudinal measures stood for a true LV dysfunction, traditional LV heart failure treatment might be of value. However, these treatments are generally not beneficial in PAH, and are often contraindicated. Risk assessment of patients with PAH has been suggested to be used at diagnosis and follow-up, where treatment escalation is recommended if a patient has not reached a low-risk status at follow-up [1, 38]. Thus, LV longitudinal measures, indicative of an underfilled LV, may add value in the risk assessment instruments.

## Limitations

Right heart catheterisation was performed on clinical indication only and therefore not performed in controls. The causality among the measures could not be proven in this cross-sectional design. Further studies with serial examinations are warranted to investigate this further.

## Conclusions

In patients with PAH, impaired LV longitudinal function was associated with low LV stroke volume and small left atrial volumes at normal left atrial pressures. This implies that an underfilling of the left heart rather than an intrinsic LV dysfunction is the mechanism behind the reduced longitudinal function. These findings were consistent irrespective of whether the underlying cause of PAH was idiopathic or associated with systemic sclerosis.

## Data Availability

All image analyses were performed in the freely available software Segment 2.2 R6887 (Medviso, Lund, Sweden; reference 18). Statistical analysis was performed using SPSS (IBM Corp. Released 2017. IBM SPSS Statistics for Macintosh, Version 25.0. Armonk, NY: IBM Corp.).
